# DEK‐targeting aptamer DTA‐64 attenuates bronchial EMT‐mediated airway remodelling by suppressing TGF‐β1/Smad, MAPK and PI3K signalling pathway in asthma

**DOI:** 10.1111/jcmm.15942

**Published:** 2020-10-30

**Authors:** Yilan Song, Zhiguang Wang, Jingzhi Jiang, Yihua Piao, Li Li, Chang Xu, Hongmei Piao, Liangchang Li, Guanghai Yan

**Affiliations:** ^1^ Jilin Key Laboratory for Immune and Targeting Research on Common Allergic Diseases Yanbian University Yanji China; ^2^ Department of Anatomy, Histology and Embryology Yanbian University Medical College Yanji China; ^3^ Postdoctoral Programme, Research Center Affiliated Hospital of Yanbian University Yanji China; ^4^ Department of Respiratory Medicine Affiliated Hospital of Yanbian University Yanji China; ^5^ Department of Intensive Care Unit Affiliated Hospital of Yanbian University Yanji China

**Keywords:** airway remodelling, aptamer, DEK, epithelial‐mesenchymal transition

## Abstract

This study is to investigate the inhibitory effects and mechanisms of DEK‐targeting aptamer (DTA‐64) on epithelial mesenchymaltransition (EMT)‐mediated airway remodelling in mice and human bronchial epithelial cell line BEAS‐2B. In the ovalbumin (OVA)‐induced asthmatic mice, DTA‐64 significantly reduced the infiltration of eosinophils and neutrophils in lung tissue, attenuated the airway resistance and the proliferation of goblet cells. In addition, DTA‐64 reduced collagen deposition, transforming growth factor 1 (TGF‐β1) level in BALF and IgE levels in serum, balanced Th1/Th2/Th17 ratio, and decreased mesenchymal proteins (vimentin and α‐SMA), as well as weekend matrix metalloproteinases (MMP‐2 and MMP‐9) and NF‐κB p65 activity. In the in vitro experiments, we used TGF‐β1 to induce EMT in the human epithelial cell line BEAS‐2B. DEK overexpression (ovDEK) or silencing (shDEK) up‐regulated or down‐regulated TGF‐β1 expression, respectively, on the contrary, TGF‐β1 exposure had no effect on DEK expression. Furthermore, ovDEK and TGF‐β1 synergistically promoted EMT, whereas shDEK significantly reduced mesenchymal markers and increased epithelial markers, thus inhibiting EMT. Additionally, shDEK inhibited key proteins in TGF‐β1‐mediated signalling pathways, including Smad2/3, Smad4, p38 MAPK, ERK1/2, JNK and PI3K/AKT/mTOR. In conclusion, the effects of DTA‐64 against EMT of asthmatic mice and BEAS‐2B might partially be achieved through suppressing TGF‐β1/Smad, MAPK and PI3K signalling pathways. DTA‐64 may be a new therapeutic option for the management of airway remodelling in asthma patients.

## 1 INTRODUCTION

Asthma is a chronic inflammatory disease characterized by the Th2/Th17 phenotypic inflammation that may cause airway structural changes termed as airway remodelling.^1^ The pathological changes of airway remodelling are characterized by epithelial damage and plasticity, increased fibroblasts/myofibroblasts, airway smooth muscle hyperplasia/hypertrophy, increased thickness of basement membrane and angiogenesis.^2^ Among them, bronchial epithelial cells undergo phenotypic changes from epithelial morphology into mesenchymal morphology, which is termed as epithelial‐mesenchymal transition (EMT).^3^ EMT is considered to be the key step of airway remodelling. During EMT, polarized bronchial epithelial cell markers such as cytokeratin and E‐cadherin are down‐regulated, and the specific markers of mesenchymal cells such as α‐smooth muscle actin (α‐SMA) and vimentin are up‐regulated.^4^ Once EMT occurs, asthma patients are not sensitive to glucocorticoids, resulting in a poor prognosis in patients. Therefore, new therapeutic options should be developed for treating asthma patients.

DEK is a sequence‐conserved nuclear protein that regulates gene expression and is considered a potential therapeutic target and biomarker for various kinds of cancers such as breast cancer, lung cancer and cervical cancer.^5^, ^6^, ^7^, ^8^, ^9^, ^10^ In triple‐negative breast cancer, DEK promoted EMT, tumour metastasis and angiogenesis through the PI3K (phosphatidylinositol 3‐kinases)/ AKT/ mTOR signalling pathway.^5^ DEK also promoted the proliferation and invasion of lung cancer cells A549 and H1299 through β‐catenin and Wnt signalling pathways.^6^ Silencing of DEK resulted in the inhibition of gastric cancer cell migration through the matrix metalloproteinase (MMP‐2/ MMP‐9) pathways.^7^ Besides, recombinant DEK promoted the proliferation of mouse hematopoietic stem cells, which was related to the enhanced activation of extracellular signal‐regulated kinases (ERK1/2), p38 mitogen‐activated protein kinases (p38 MAPK) and AKT signalling pathways.^8^, ^9^ However, silencing of DEK in cervical cancer regulates tumorigenesis and metastasis via enhancing GSK‐3β activity, while down‐regulating Wnt/β‐catenin and MMP‐9.^10^ Recently, it has been shown that DEK is a pro‐inflammatory factor that mediates neutrophil inflammation.^11^, ^12^ In juvenile arthritis patients, DEK is found in the neutrophil extracellular traps (NETs) of the synovial fluid, and targeting DEK can reduce the formation of NETs and joint inflammation.^11^ In addition to pro‐inflammatory effects, DEK also has a pro‐inflammatory chemotactic function. In response to interleukin 8 (IL‐8), DEK is actively secreted by macrophages in free form or exosomes, which then attracts neutrophils, natural killer cells and CD8+ T cells.^12^ Therefore, DEK may be involved in the development of inflammation and tumorigenesis and is thus suitable as a therapeutic target. However, there is no evidence that DEK plays a direct role in allergic airway inflammation.

Aptamers are small, single‐stranded oligonucleotides with high binding specificity and affinity to target proteins as a result of their ability to folds into 3D scaffolds as antibody‐like binding and are often called as ‘chemical antibodies’.^13^ Since they are easily chemically modified, synthesized and highly stable, they are beneficial for targeted therapeutics and diagnosis. They can act as antagonists and have been used in clinical trials, such as CCL2‐targeting aptamer NOX‐E36 Noxxon Pharma in the chronic inflammatory disease, type 2 diabetes, systemic lupus, albuminuria and other inflammatory diseases.^13^ In this study, we used DEK‐targeting aptamer as DEK inhibitor and investigated its role in airway inflammation and EMT in OVA (ovalbumin)‐induced mouse model of asthma. The underlying mechanisms involving TGF‐β1/Smad, MAPK and PI3K signalling pathways were also analysed and discussed.

## 2 MATERIALS AND METHODS

### 2.1 Mice

Seven‐week‐old female BALB/c mice (weight 20‐22 g) were purchased from HOUSE section of Yanbian University Health Science Center (Yanji, China). All mice were housed in the specific pathogen‐free condition with room temperature of 22 ± 2°C, relative humidity of 50%‐60% and 12‐hour light‐dark cycle. The animal experiment procedures were approved by the Institutional Animal Care and Use Committee of Yanbian University (approval no. SCXK (JI)2017‐0003).

### 2.2 OVA‐induced asthmatic mouse model

**FIGURE 1:**
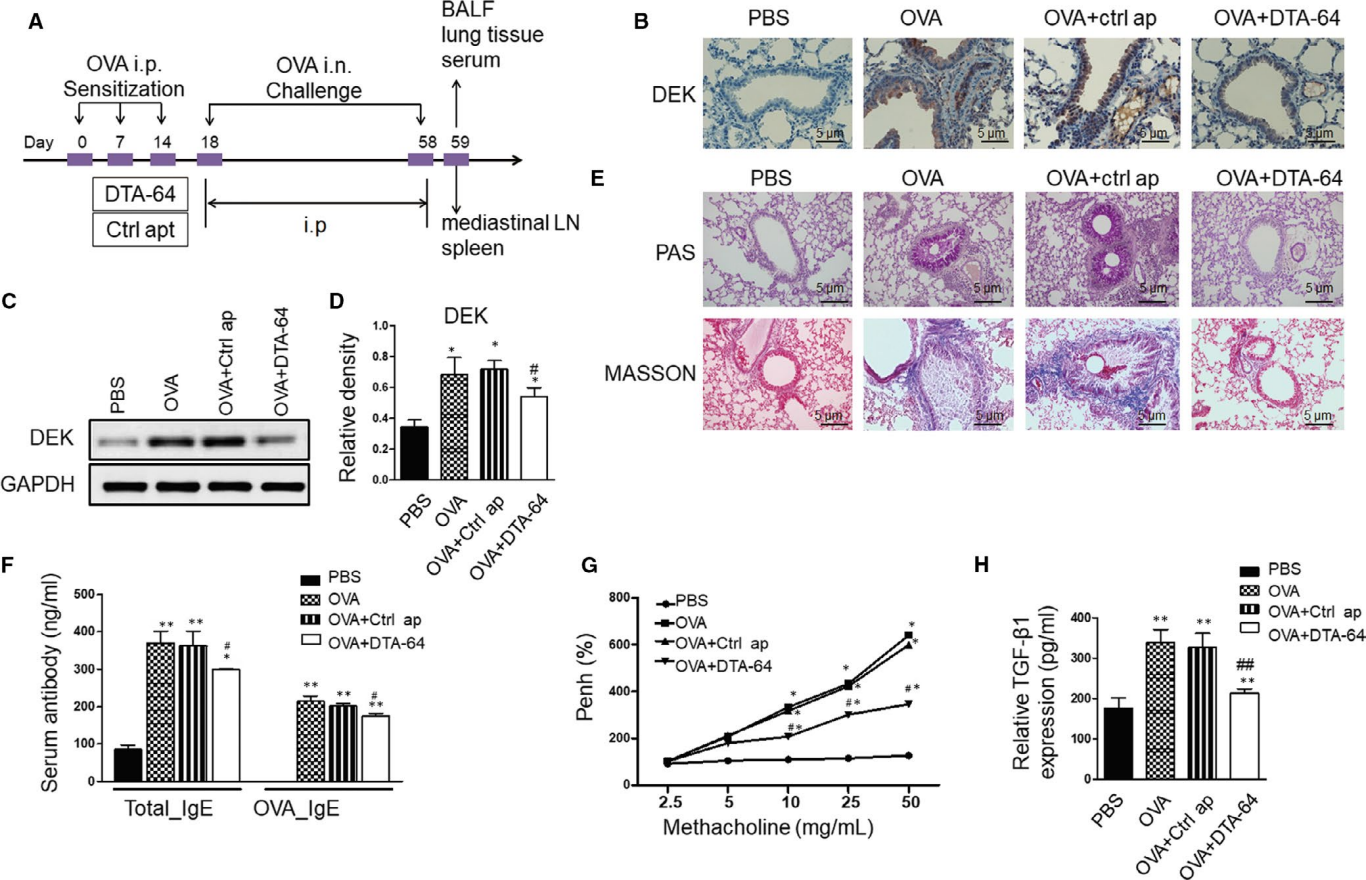
Effects of DTA‐64 on inflammatory cell infiltration, lung histology, IgE and AHR. (A) Schematic diagram of the experimental protocol. (B) Immunohistochemical staining was performed to detect the production of DEK (×200 magnification, scale bar: 5 μm). (C) Western blot of DEK in the lung homogenates. (D) Relative expression of DEK. (E) PAS staining for measurement of mucus production and Masson staining for collagen deposition around the airways (×200 magnification, scale bar: 5 μm). (F) The concentrations of total IgE and OVA‐specific IgE were detected by ELISA. (G) AHR was measured after treating with methacholine. (H) Relative TGF‐β1 level analysed by ELISA. All data were shown as mean ± SD (n = 3). ^*^ *P* < 0.05, ^**^ *P* < 0.01 vs control group. ^#^ *P* < 0.05, ^##^ *P* < 0.01 vs OVA group

The schematic diagram of the experimental protocol is shown in Figure 1A. Briefly, mice were sensitized on days 0, 7 and 14 by intraperitoneal injection (*i.p*.) with 10 μg of OVA(Sigma‐Aldrich, St. Louis, MO, USA) and 1 mg of aluminium hydroxide adjuvant (Invivo Gen, San Diego, CA, USA), which were dissolved in 200 μL PBS. Control mice were *i.p*. injected by an equivalent amount of PBS. Mice were then inhaled with 5% OVA in sterile PBS for 30 min and three times a week for 8 weeks. Mice were divided into PBS group, OVA group, OVA + Control aptamer group and OVA + DTA 64 (DEK‐targeting aptamer) group, with 7 mice in each group. For mice in OVA + Control aptamer group and OVA + DTA 64 group, 1 μg control aptamers or DTA 64 dissolved in 200 μL PBS were injected intraperitoneally after OVA sensitization from day 18, as previously described.^11^ After 8 weeks of OVA inhalation, all mice were euthanized with 100 mg/kg pentobarbital sodium. Aptamers were synthesized from ‘Systematic evolution of ligands by exponential enrichment’ (SELEX) process and purified using high‐performance liquid chromatography at a purity of 99% (Sangon Biotech, Shanghai, China).

### 2.3 Enzyme‐linked immunosorbent assay

Total and OVA‐specific immunoglobulin E (IgE) in serum, and IL‐4, IL‐17, IFN‐γ and TGF‐β1 in bronchoalveolar lavage fluid (BALF) were determined using corresponding mouse ELISA Kits (R&D Systems, Minneapolis, MN, USA). The sensitivity for IL‐4, IL‐17, TGF‐β1 and IFN‐γ were all 2.0 pg/mL.

### 2.4 Evaluations of AHR

After the final OVA inhalation challenge, lung resistance of asthmatic mice was measured with flexi Vent forced oscillation technique (SCIREQ, Montreal, Canada) to evaluate AHR. Briefly, mice were anesthetized using pentobarbital sodium (100 mg/kg), and then, the trachea was exposed followed by insertion of a cannula for administration with aerosol methacholine (2.5, 5, 10, 25, 50 mg/mL). The lung resistance was measured to evaluate the AHR quantitatively.

### 2.5 Histological analysis of lung tissue

The lung tissues were fixed with 4% paraformaldehyde for 24 hours, embedded in paraffin and cut into 4‐μm sections. The sections were stained with haematoxylin‐eosin (H&E), Periodic Acid‐Schiff (PAS) and Masson. For immunohistochemistry staining, lung sections were incubated with rabbit anti‐mouse DEK antibody (#74975, 1:200; Abcam, Cambridge, MA, USA) and then incubated with goat anti‐rabbit IgG H&L (HRP) (#6721, 1:2000; Abcam). Each section was randomly selected, and in total, five different fields were photographed under a light microscope (Eclipse Ni‐U, Nikon, Japan) at 200× magnification.

### 2.6 Single‐cell suspension of the lung, mediastinal lymph node and spleen

To detect the production of α‐SMA in the lung, lung tissues were incubated with 1 mg/mL Collagenase A (#17100017; Gibco, Gaithersburg, MD, USA), 1500 kU/mL DNase I (#D5025‐150KU; Sigma‐Aldrich Chemical Co., St. Louis, MO, USA) and CaCl_2_ (0.025M) at 37℃ for 4 hours to prepare single cells suspension, as previously reported.^14^ For detecting the cytokines levels in the mediastinal lymph node (mLN) and spleen, lymph nodes and spleen were isolated and grinded with ground glasses, and cells were prepared for further detection by flow cytometry.

### 2.7 Flow cytometry

For surface molecular staining, obtained cells were incubated with APC‐conjugated CD45.2 antibody (#558702; BD Biosciences, San Jose, CA, USA), Percp‐Cy5.5‐conjugated CD3e antibody (#45‐0031‐82; Invitrogen, Carlsbad, CA, USA ), FITC‐conjugated CD4 antibody (#11‐0041‐82; Invitrogen), APC‐Cy7‐conjugated Ly‐6G antibody (#560600; BD), PE‐conjugated Siglec‐F antibody (#552126; BD), PE‐CY7‐conjugated CD31 (#561410; BD) and PE‐conjugated EpCAM (#563477; BD). For intracellular staining, the Intracellular Fixation &Permeabilization kit (#88‐8824‐00; Invitrogen Co, Carlsbad, CA, USA) was used. The antibodies used were as follows: PE‐Cy7‐conjugated IL‐4 antibody (#25‐7042‐41; Invitrogen), APC‐conjugated IFN‐γ antibody (#17‐7319‐41; Invitrogen) and PE‐conjugated IL‐17 antibody (#561020, BD). Primary antibodies for intracellular staining included α‐SMA (#7817; Abcam), TGF‐β1 (#92486; Abcam), DEK (#166624; Abcam), E‐cadherin (#40772; Abcam) and Vimentin (#8978; Abcam). After that, incubation with Alexa Fluor 488 goat anti‐rabbit or anti‐mouse IgG secondary antibody (Invitrogen, #1810918, #2018309) was performed. The CD45.2^‐^CD31^‐^EpCAM^+^ cells were gated for lung epithelial cells and analysed.

### 2.8 Cell line and cell treatment

Human bronchial epithelial cell line BEAS‐2B was purchased from American Type Culture Collection (Rockville, MD, USA). DEK or DEK shRNA containing plasmids were synthesized by Shanghai GeneChem (Shanghai, China). Plasmids of DEK overexpression (ovDEK), empty vector (NC), DEK shRNA (shDEK) and a scrambled shRNA (shNC) were, respectively, transfected into the BEAS‐2B cells using Lipofectamine 3000 (Invitrogen) according to manufacturer's instructions. After culturing cells in the absence of FBS for 18 hours, cells were stimulated with 10 ng/mL TGF‐β1 in 10% FBS for 24 hours.

### 2.9 Western blot

Total, cytosolic and nuclear proteins were freshly isolated from lung tissues using Nuclear Protein Extraction Kit (Solarbio, China). The protein concentrations were determined using BCA protein assay kit (Beyotime, China). Then, 30 μg of protein samples were separated using 12% SDS‐PAGE and transferred to nitrocellulose membranes. After blocking with 5% skimmed milk, the membranes were incubated with primary antibodies at 4°C overnight and then incubated with goat anti‐rabbit secondary antibody (#5151) or anti‐mouse secondary antibody (#5257; Cell Signaling Technology (CST, Danvers, MA, USA)) for 2 hours at room temperature. The primary antibodies of IκBα (#4814), phospho (p)‐IκBα (#2859), p‐ERK (#4370), ERK (#4695), Jun N‐terminal kinases (JNK) (#9253), p‐JNK (#9255), p38 (#8690), p‐p38 (#4511) and GAPDH (#2118) were purchased from CST; those of NF‐κB p65 (#207297), PI3K (#18705), PARP (#74290), p‐AKT (#38449), AKT (#38449), p‐mTOR (#109268), mTOR (#32028), TGF‐β1 (#92486), p‐Smad2/3 (#ab63399), Smad2/3 (ab202445), Smad4 (ab230815), MMP‐2 (#92536), MMP‐9 (#38898), snail + slug (#180714), E‐cadherin (#40772), Vimentin (#8978) and α‐SMA (#7817) were purchased from Abcam. ECL detection reagent (Beyotime Biotechnology, China) was used for colour development. Quantity One (Bio‐Rad, Hercules, CA, USA) was used for analysing grey density. Relative protein levels were calculated as the ratio of grey density.

### 2.10 RT‐PCR

Total RNAs from BEAS‐2B cells were extracted using RNA Easy Fast kit (Tiangen, Beijing, China), and 2 μg of total RNA was reverse transcribed into cDNA with Fast King RT kit (Tiangen). The cDNA (50 ng) was used as template for RT‐PCR. RT‐PCR was performed using two‐step SYBR green qPCR assays (Transgene, Biotech, G31227), and the target genes were amplified using following specific primers: α‐SMA forward: GTGTTGCCCCTGAAGAGCAT, reverse: GCT GGGACATTGAAAGTCTCA; E‐cadherin forward: TTCCCTGCGTATACCCTGGT, reverse: GCGAAGATACCGGGGGACACTCATGAG; Vimentin forward: AGGAA ATGGCTCGTCACCTTCGTGAATA, reverse: GGAGTGTCGGTTGTTAAGAACT AGAGCT; GAPDH forward: GAGTCAACGGATTTGGTCGT, reverse: TTGATTTT GGAGGGATCTC. All values were finally normalized to β‐actin transcript levels.

### 2.11 Immunofluorescence

Lung sections and BEAS‐2B cells were prepared and incubated with the primary antibodies of NF‐κB (#207297, Abcam), α‐SMA (#7817, Abcam), E‐cadherin (#40772, Abcam) and Vimentin (#8978, Abcam) at 4°C overnight and then incubated with Alexa Fluor 488 goat anti‐rabbit or anti‐mouse IgG secondary antibody (Invitrogen, #1810918, #2018309) for 2 hours. The samples were further observed using Cytation™ 5 (BioTek Instruments, Inc., Winooski, VT, USA).

### 2.12 Statistical analysis

Significant differences between the groups were analysed using ANOVA or Wilcoxon's rank‐sum test. All data were shown as mean ± standard deviation (SD), and a value *P* < 0.05 was considered as statistically significant.

## 3 RESULTS

### 3.1 DTA‐64‐treated mice have less asthmatic inflammation, AHR, IgE production and lung histological damage in response to OVA

DTA‐64 is generated using SELEX technology and a single‐stranded DNA aptamer containing 41 nucleotide cores. It has high combining ability to the recombinant DEK protein and has inhibitory effect on DEK.^11^ To test the effect of DTA‐64 on OVA‐induced asthma, we treated asthma mice with different strategies and then evaluated the severity of asthma inflammation by pathological assessment. Immunohistochemical staining and Western blot of lung tissues showed that OVA could induce DEK in the airway of asthmatic mice (Figure 1B‐D). However, this induction was inhibited in the DTA‐64‐treated group (Figure 1B‐D). Besides, PAS staining showed that PAS‐positive cells in the airway epithelium of OVA + DTA‐64 group were greatly lower than those of the OVA group (Figure 1E). Masson's trichrome staining showed that compared with the OVA group, collagen deposition was significantly relieved in DTA‐64‐treated mice (Figure 1E). As shown in Figure 1F, serum OVA‐specific IgE levels in the OVA group increased significantly, and however, this was suppressed by DTA‐64 treatment. In addition, compared to methacholine inhalation in PBS and control aptamer groups, a lower methacholine inhalation was induced by DTA‐64 treatment (Figure 1G). Furthermore, we also detected the TGF‐β1 expression in the BALF and found that levels of TGF‐β1 were decreased in DTA‐64 group compared with both OVA group and control aptamer group (Figure 1H). Therefore, all these results indicate that DTA‐64 can reduce OVA‐induced asthma by reducing goblet cell hyperplasia, TGF‐β1 expression, serum IgE and AHR.

### 3.2 DTA‐64 decreases inflammatory cell infiltration in BALF and NF‐κB activation in the lung

**FIGURE 2:**
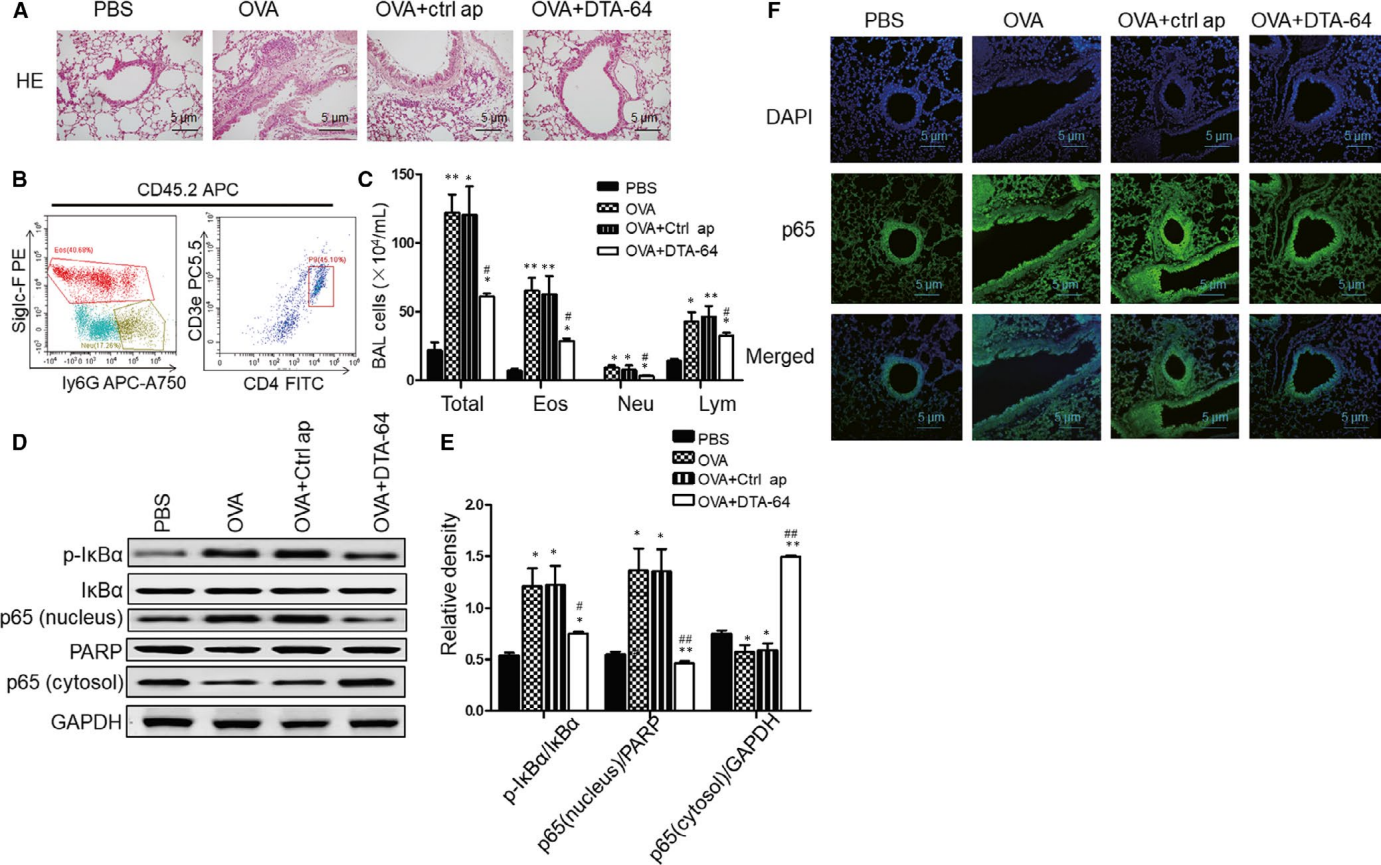
Effects of DTA‐64 inflammatory cells infiltration and NF‐κB signalling pathway in the lung. (A) H&E staining for inflammatory cell infiltration (×200 magnification, scale bar: 5μm). (B) The gating strategy of total cells, eosinophils and neutrophils in the BALF. Granulocytes in the BALF were gated based on the CD45.2 + properties. Within the CD45.2 gate, CD45.2+/CD11b+/ly6G+, CD45.2+/Siglec‐F + cells and CD3e+/CD4+ cells represented the eosinophils, neutrophils and T cells, respectively. (C) The numbers of total cells, eosinophil (EOS), neutrophil (Neu) and T cells (Lym). (D) The production of NF‐κB p65 (nuclear), NF‐κB (cytosol), p‐IκBα and IκBα in the lung homogenates was estimated by western blotting, and the relative density (E) was measured. (F) Immunofluorescence assay for NF‐κB p65 in the lung sections was performed. All data were shown as mean ± SD (n = 3). ^*^ *P* < 0.05, ^**^ *P* < 0.01 vs control group. ^#^ *P* < 0.05, ^##^ *P* < 0.01 vs OVA group

Next, we examined the effects of DTA‐64 on inflammatory cell infiltration and NF‐κB activation in the lung. H&E staining of lung showed that DTA‐64 treatment greatly alleviated inflammatory cell infiltration (Figure 2A). Besides, flow cytometry showed that compared to the OVA‐challenged group, there was a dramatic decrease of total cells, neutrophils (CD45.2+/CD11b+/ly6G+), eosinophils (CD45.2+/siglec‐F+) and CD4^+^ cells (CD3e+/CD4+) in the BALF after DTA‐64 treatment (Figure 2B and C). On the other hand, OVA challenge promoted NF‐κB p65 translocation from cytosol to nuclei, and DTA‐64 led to suppression of nuclear translocation of p65 subunit in lung tissues (Figure 2D and E). Furthermore, the effects of DTA‐64 on OVA‐induced phosphorylation and degradation of IκBα were also detected to verify the mechanisms of DTA‐64 on NF‐κB p65 nuclear translocation. Indeed, DTA‐64 greatly decreased the phosphorylation and degradation of IκBα in the cytosol of lung tissues (Figure 2D and E). Similarly, using immunofluorescence, we found that the NF‐κB p65 in the lung sections was significantly decreased in the OVA + DTA‐64 group compared to the OVA and OVA + control aptamers group (Figure 2F). These results reveal that administration of DTA‐64 could efficiently decrease the inflammatory cell migration and prevent nuclear translocation of NF‐κB p65 through inhibiting phosphorylation and degradation of IκBα in asthmatic mice.

### 3.3 Immunomodulatory effects of DTA‐64 on Th1/Th2 balance in OVA‐induced asthmatic mice

**FIGURE 3:**
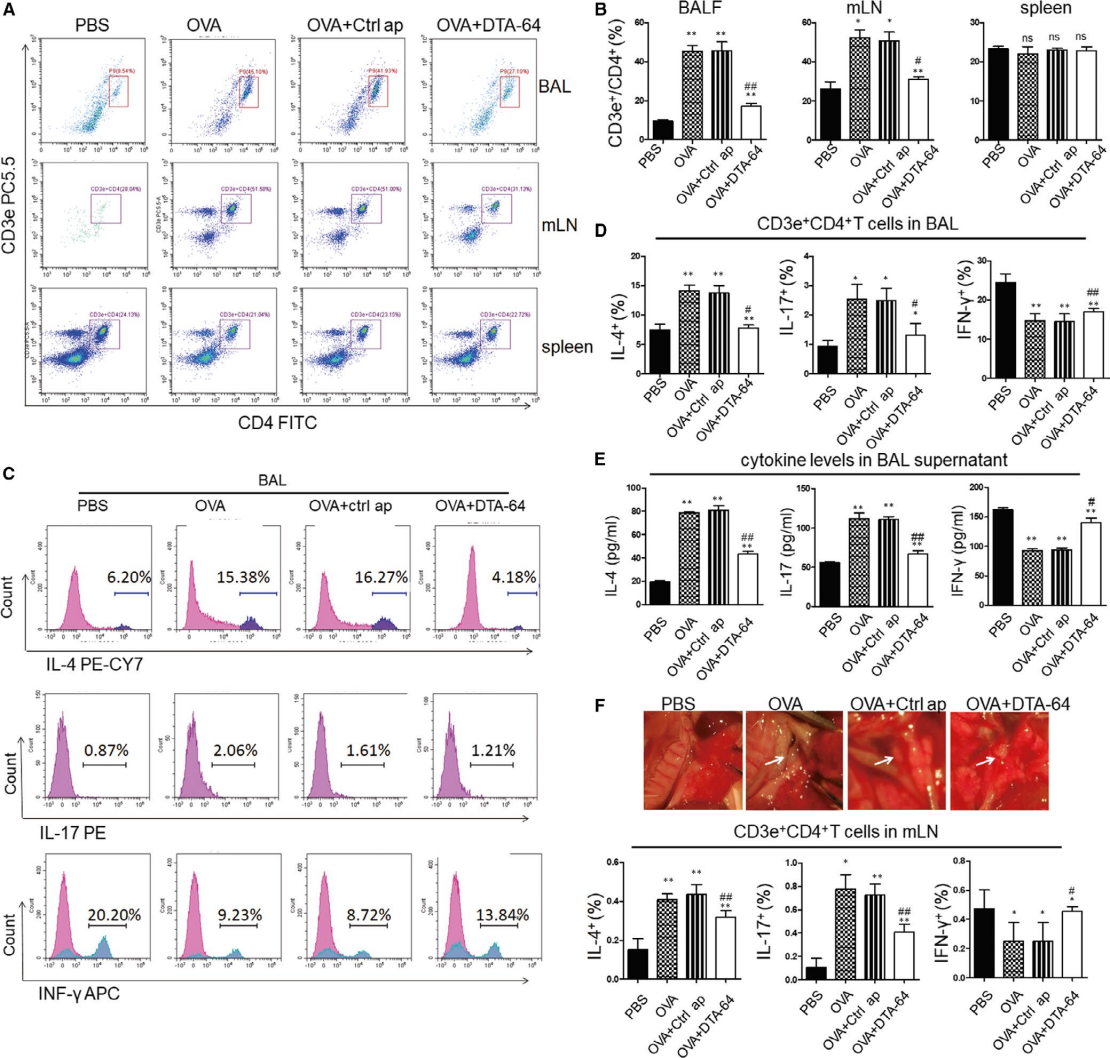
Effects of DTA‐64 on the Th1/Th2/Th17 balance in vivo. Flow cytometric analysis of CD4^+^T cell and Th1/Th2/Th17 subtypes was performed in BAL cells, mLNs and splenocytes. (A) CD3e+/CD4+ cells in the BAL cells, mLNs and splenocytes were selected. (B) Proportion of CD3e+/CD4+ cells was displayed by histogram. (C) Th1/Th2/Th17 cytokines within the CD3e+ CD4+ cells in the BAL cells were selected. (D) Levels of IL‐4, IL‐17 or IFN‐γ secreting cells within the CD3e+/CD4+ cells were shown by histogram. (E) Production of IL‐4, IL‐17 and IFN‐γ was measured by ELISA. (F) The photograph of mLNs in the group mice. Arrows indicate the mLN. (G) Proportion of IL‐4, IL‐17 and IFN‐γ‐positive cells in the CD3e+/CD4+ cells were displayed by histogram. All data were shown as mean ± SD ^*^ *P* < 0.05, ^**^ *P* < 0.01 vs control group. ^#^ *P* < .05, ^##^ *P* < 0.01 vs OVA group

Asthma is a Th2‐driven disease and always characterized by the imbalance among the Th1, Th2 and Th17 immune responses.^15^ To investigate whether the DTA‐64 affects the Th immune response in the OVA‐challenged mice, we detected the subtypes of CD4^+^ T cells and Th1/Th2/Th17 cytokines in the BAL cells, mLN and spleen. The results showed that the CD4^+^ T cells (CD3e+/CD4+) increased in the BAL cells and mLNs, but not in the spleen of OVA‐challenged mice (Figure 3A and B). However, DTA‐64 treatment could decrease the elevation of CD4^+^ T cells compared with the OVA group. Besides, IL‐4 and IL‐17, which, respectively, indicate the T2/Th17 immune response, were significantly decreased in the DTA‐64 group, while IFN‐γ that represents the Th1 immune response was increased (0.5‐fold) in the BAL cells after DTA‐64 treatment when compared with OVA group (Figure 3C and D). Similarly, ELISA also showed that cytokine levels of IL‐4 and IL‐17 in the BAL supernatants were significantly decreased and IFN‐γ was significantly increased in the DTA‐64 administration group when compared to the OVA group (Figure 3E). In addition, we found enlarged mLN in the OVA group compared with control group, and DTA‐64 treatment reduced the size of mLN (Figure 3F). We also found that DTA‐64 also had similar regulatory effect on the Th imbalance in the mLN cells as that in BAL cells (Figure 3G). Taken together, these results indicate that DTA‐64 has immunomodulatory effects on the differentiation of CD4^+^ T cells, which is biased to Th1 cells.

### 3.4 DTA‐64 inhibits α‐SMA production in the lung epithelial cells

**FIGURE 4:**
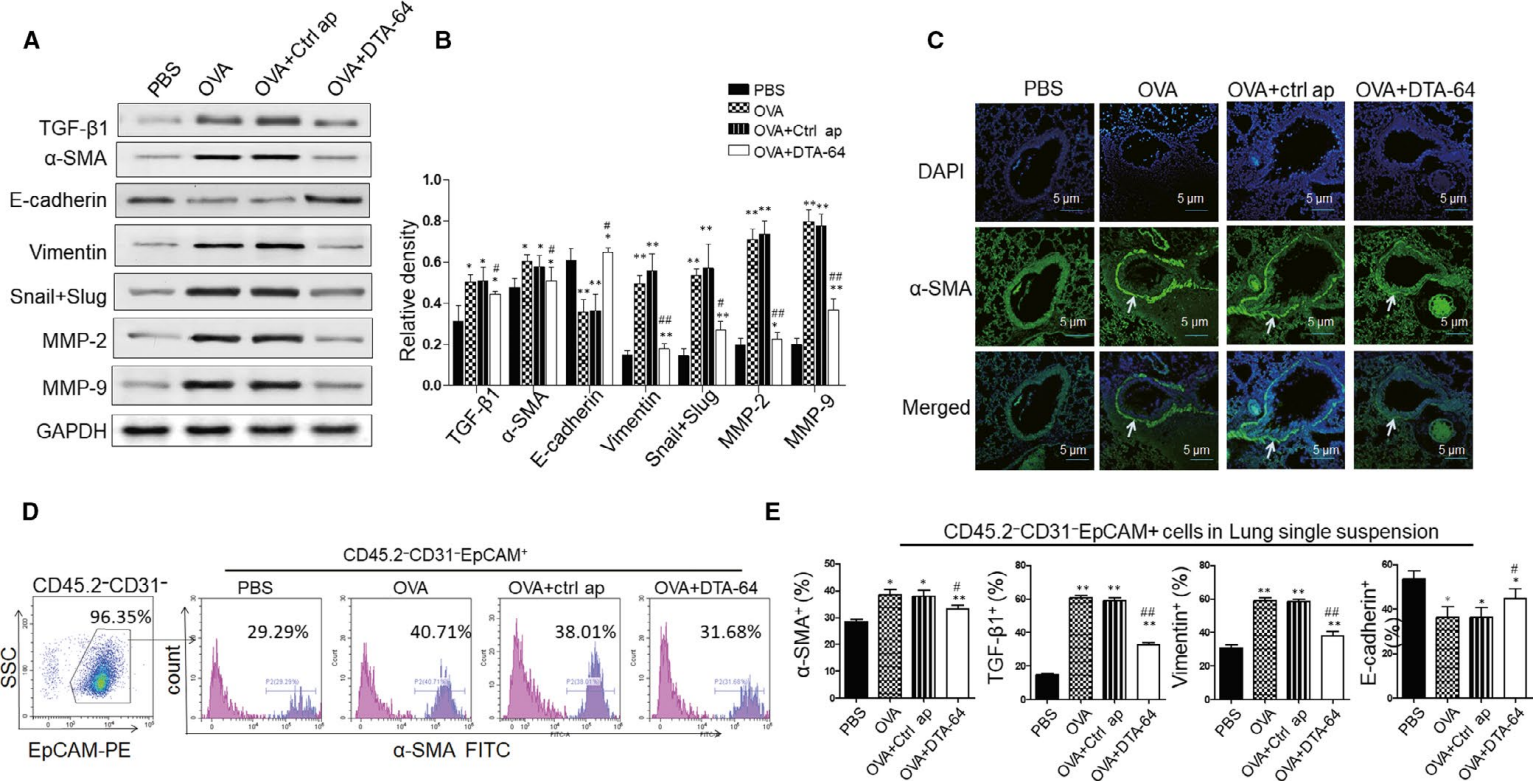
DTA‐64 inhibits α‐SMA production in the lung epithelial cells. (A) The α‐SMA, vimentin, slug, snail MMP‐2, MMP‐9 and E‐cadherin were detected by Western blotting. (B) Relative density of the above proteins in the lung homogenates. (C) The production of α‐SMA was measured in the lung section by immunofluorescence assay. CD45.2^‐^ cells were gated for α‐SMA measurement (arrow). (D) Gating strategy of α‐SMA, TGF‐β1, vimentin, E‐cadherin in the lung epithelial cells using CD45.2^‐^ (immune cell marker) and CD31^‐^ (endothelial marker), EpCAM^+^ (epithelial cell adhesion molecule marker) by flow cytometry. (E) Proportions of α‐SMA, TGF‐β1, vimentin and E‐cadherin in the CD45.2^‐^CD31^‐^EpCAM^+^ lung epithelial cells were shown. All data were shown as mean ± SD (n = 3). ^*^ *P* < 0.05, ^**^ *P* < 0.01 vs control group. ^#^ *P* < 0.05, ^##^ *P* < 0.01 vs OVA group

To evaluate the effect of DTA‐64 on the EMT in the lung tissue of asthmatic mice, we detected TGF‐β1 and mesenchymal markers (α‐SMA, vimentin, slug, snail) and matrix metalloproteins (MMP‐2 and MMP‐9), as well as epithelial marker E‐cadherin in the lung homogenates. We found that mice treated with DTA‐64 showed a decrease of TGF‐β1, α‐SMA, slug, snail, vimentin, MMP‐2 and MMP‐9, while an increase of E‐cadherin (Figure 4A and B). We then further validated level of α‐SMA in the lung sections using immunofluorescence and found that α‐SMA was greatly elevated in the OVA‐induced asthmatic mice and was dramatically down‐regulated by the DTA‐64 (Figure 4C). Furthermore, we also detected α‐SMA and TGF‐β1, vimentin, E‐cadherin in the lung epithelial cells using negative selection of CD45.2 (immune cell marker) and CD31 (endothelial marker), positive selection of epithelial cell adhesion molecule (EpCAM) by flow cytometry. The results showed that DTA‐64 down‐regulated α‐SMA, TGF‐β1, vimentin and up‐regulated E‐cadherin in the CD45.2^‐^CD31^‐^EpCAM^+^ lung epithelial cells when compared with OVA group (Figure 4D and E). These findings imply that DEK inhibition using DTA‐64 inhibits the progression of EMT in the lung.

### 3.5 DEK silencing inhibits the progression of EMT in human bronchial epithelial cell

**FIGURE 5:**
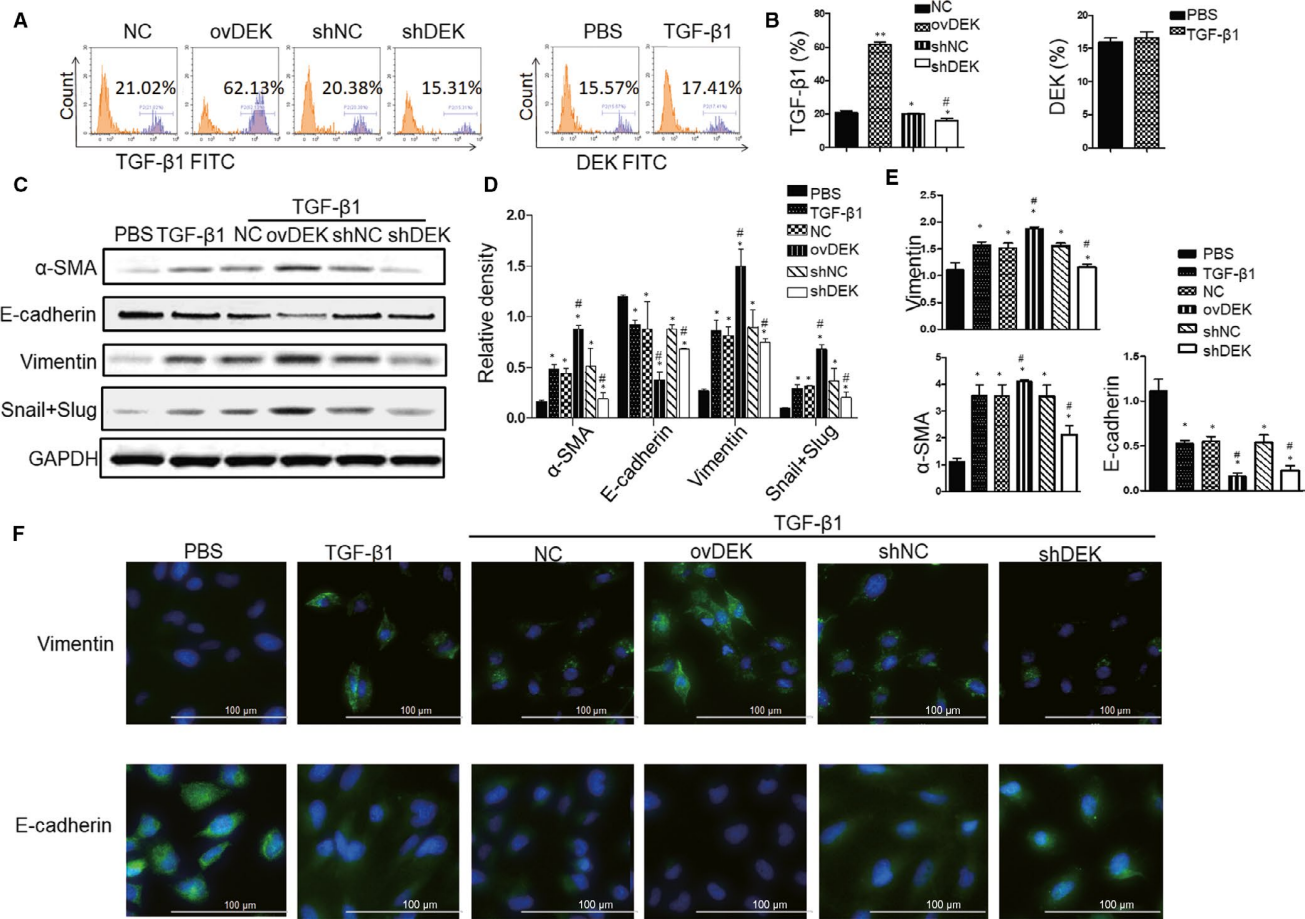
DEK positively regulates the progression of EMT in human bronchial epithelial cell. (A) Flow cytometry analysis of TGF‐β1 expression in the BEAS‐2B cells with ovDEK or shDEK (right panel) and DEK expression after treating with TGF‐β1 (left panel). (B) Relative level of TGF‐β1 and DEK. (C) The DEK, α‐SMA, vimentin, E‐cadherin and Snail + Slug were detected after TGF‐β1 treatment using Western blotting. (D) Relative density of above proteins. (E) mRNA levels of α‐SMA, vimentin and E‐cadherin were detected. (F) Immunofluorescence was performed for analysis of E‐cadherin and vimentin in the BEAS‐2B cells. NC, negative control cells; ovDEK, cells transfected with DEK; shNC, cells transfected with scrambled shRNA; shDEK, cells transfected with DEK shRNA.^*^ *P* < 005, vs control group. ^#^ *P* < 0.05, ovDEK vs TGF‐β1 + NC group; shDEK *v s*TGF‐β1 + shNC group

In order to determine the effect of DEK on the EMT process in the human bronchial epithelial cells (BEAS‐2B), a cell line widely used to study EMT,^16^ we respectively transfected cells with DEK overexpressing (ovDEK) or silence (shEDK) plasmids. We then measured E‐cadherin, α‐SMA and vimentin after treating with TGF‐β1. Based on the in vivo experiments that administration of DTA‐64 could down‐regulate TGF‐β1 expression in BALF, we firstly detected the TGF‐β1 expression in the BEAS‐2B cells with ovDEK or shDEK and found that TGF‐β1 increased threefold in the ovDEK group, slightly decreased in shDEK group when compared with NC group; on the contrary, DEK expression was not affected by TGF‐β1 exposure (Figure 5A and B). Moreover, cells transfected with ovDEK exhibited higher mRNA or protein levels of α‐SMA and vimentin and lower levels of E‐cadherin compared with TGF‐β1 group and TGF‐β1 + NC alone (Figure 5C, D and E). However, cells transfected with shDEK had the opposite effects compared to the TGF‐β1 + shNC. Similarly, by further using immunofluorescence, we obtained the similar results, which showed that cells overexpressing DEK had higher vimentin and lower E‐cadherin than that of TGF‐β1 or TGF‐β1 + NC cells (Figure 5F). However, silencing of DEK had lower vimentin and higher E‐cadherin than that of TGF‐β1 + shNC cells. These data suggest that DEK overexpression promotes EMT progression induced by TGF‐β1 in the BEAS‐2B cells, whereas silencing of DEK inhibits the progression of EMT.

### 3.6 DEK positively regulates the progression of EMT in human bronchial epithelial cell

**FIGURE 6:**
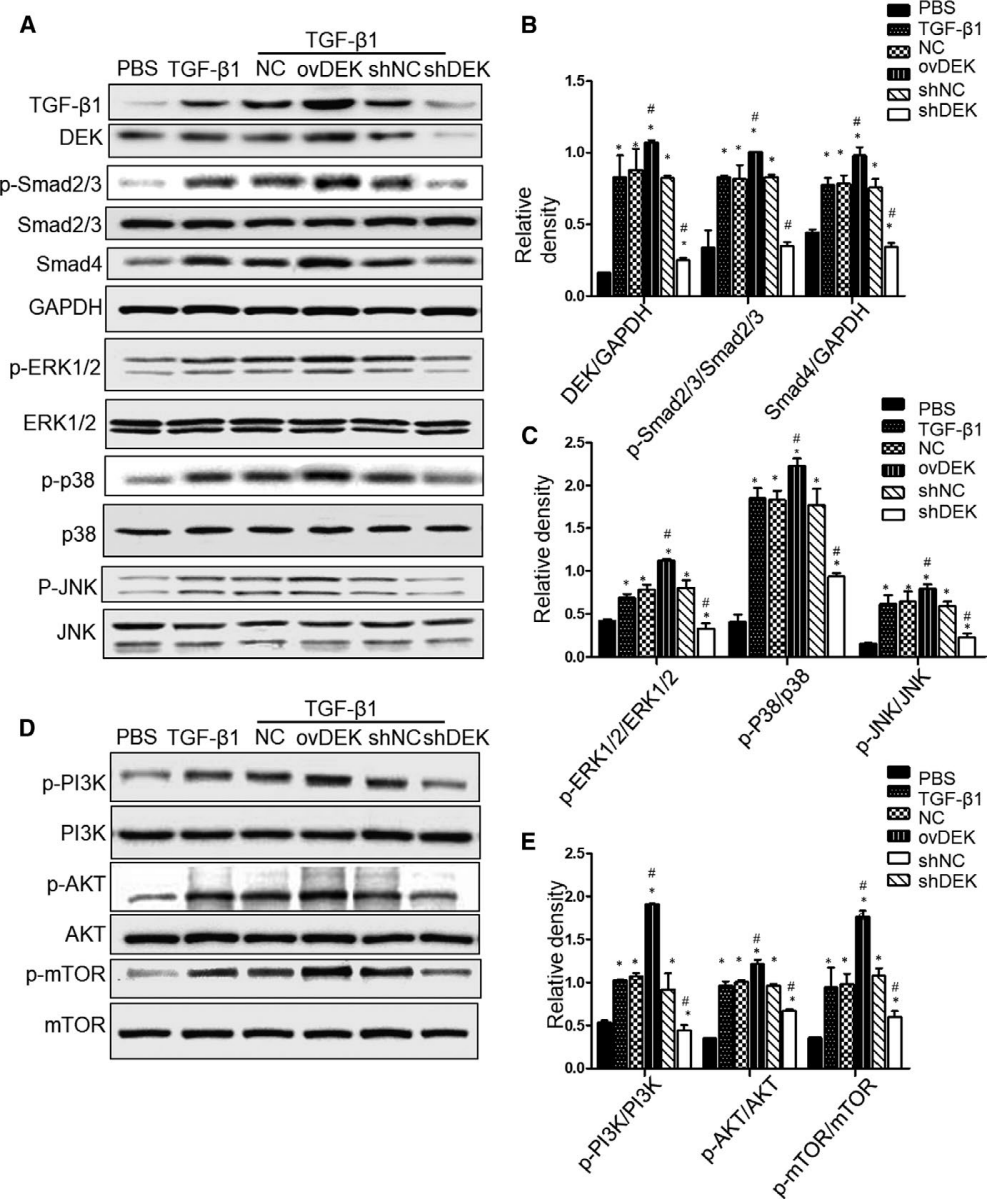
Effects of DEK shRNA on Smad, MAPK and PI3K/AKT signalling pathways in BEAS‐2B cells. (A‐E) The expressions of TGF‐β, DEK mad2/3 and Smad4, ERK1/2, p38, JNK, and PI3K, AKT, mTOR were detected by Western blotting. The relative density of each protein was calculated. Data were shown as mean ± SD (n = 3). NC, negative control cells; ovDEK, cells transfected with DEK; shNC, cells transfected with scrambled shRNA; shDEK, cells transfected with DEK shRNA. ^*^ *P* < 0.05, vs control group. ^#^ *P* < 0.05, ovDEK vs TGF‐β1 + NC group; shDEK vs TGF‐β1 + shNC group

To investigate the mechanisms involved in the EMT modulation by DEK overexpression or silencing in BEAS‐2B cells, we detected phosphorylation of key components in the signal transduction pathways, including Smad, MAPK and PI3K/AKT pathways. Interestingly, ovDEK group could promote TGF‐β1 production, and shDEK group showed opposite effects on TGF‐β1 production (Figure 6A). Moreover, TGF‐β1 stimulation could not induce DEK production. Continuously, ovDEK group exhibited higher Smad 2/3 phosphorylation and Smad4, and DEK silencing showed lower Smad 2/3 phosphorylation and Smad 4 after TGF‐β1‐mediated EMT in the BEAS‐2B cells (Figure 6A and B). Additionally, DEK overexpression or DEK shRNA have the similar effects on the TGF‐β1‐mediated non‐canonical pathway of MAPK pathway, including ERK1/2, p38 MAPK and JNK, by up‐regulating their phosphorylation rate for around 20%‐25% or down‐regulating their phosphorylation rate for approximately 40%‐55%, respectively (Figure 6A and C). We also examined the role of DEK in the PI3K/AKT/mTOR signalling pathway, which is also involved in the TGF‐β1‐mediated non‐canonical pathway of EMT. Our data indicated that cells overexpressing DEK induced phosphorylation of PI3K, AKT, and mTOR, whereas cells with silenced DEK exhibited totally blocked phosphorylation of PI3K, AKT and mTOR in BEAS‐2B cell (Figure 6D and E). Accordingly, these findings indicate that DEK may affect EMT likely through regulating Smad, MAPK and PI3K/AKT signalling pathways.

## 4 DISCUSSION

DEK is highly expressed in inflammatory diseases and tumours. It is usually found in the nucleus and is released by macrophages, T cells and neutrophils.^9^ However, the role of DEK in bronchial asthma is rarely reported. In this study, we first revealed the importance of DEK for the development of bronchial asthma in vivo and in vitro. Using a mouse model of asthma induced by OVA, we found that DEK was overexpressed in lung sections of asthmatic mice and that DTA‐64 (single‐stranded DNA aptamers targeting DEK) significantly reduced airway remodelling, including infiltration of inflammatory cells, airway mucus production, goblet cell proliferation, BALF TGF‐β1 expression, serum IgE, AHR and EMT. We also found that DTA‐64 treatment greatly attenuated the levels of Th2 and Th17 cytokines (such as IL‐4 and IL‐17) and up‐regulated Th1 cytokines IFN‐γ. In the in vitro setting, we found that DEK overexpression promoted EMT, while interference of DEK inhibited EMT in the BEAS‐2B cells. These anti‐airway remodelling effects of DTA‐64 are supported by previous study, in which DEK was very successfully suppressed in DTA‐64‐treated arthritis model mice.^11^ Therefore, DEK may be a potential therapeutic target for OVA‐induced bronchial asthma.

NF‐κB signalling pathway can regulate airway remodelling ^17^ and can mediate the EMT of pancreatic cancer cell.^18^ Here, we found that the lung tissue of mice challenged with OVA showed obvious enhancement of NF‐κB, IκB phosphorylation and P65 translocation, while this was significantly inhibited after DTA‐64 treatment. These results were supported by a previous report that DEK promoted inflammation through NF‐κB pathway,^19^ indicating that the inhibition of DEK on inflammatory response might be due to the inhibition of the NF‐κB signalling pathway in OVA‐challenged asthmatic mice.

Allergic asthma usually manifests as an imbalance between Th1 and Th2 immune responses.^20^, ^21^ Early studies in patients with mild asthma have shown that CD4^+^ T cells favour Th2 type immune responses in BALF, lung tissue, and blood, and secrete large amounts of cytokines, such as IL‐4, IL‐5, IL‐13 and IL‐9, while Th1‐type cytokines such as IFN‐γ, IL‐2 and IL‐12 are reduced.^22^, ^23^ In addition, IL‐4, IL‐5 and IL‐13 block the production of IFN‐γ, which inhibits the activation of eosinophils, basophils and mast cells.^24^ Mice lacking Th2 cytokines have a significant reduction in inflammation in asthma mice challenged with OVA.^25^ In our study, DTA‐64 significantly attenuated the levels of Th2 and Th17 cytokines such as IL‐4 and 17, and promoted the secretion of Th1 cytokine IFN‐γ. In addition, in mice treated with DTA‐64, the ratio of Th1/Th2 was significantly reduced. These results confirm that DEK‐targeted aptamers can affect Th1/Th2 balance in bronchial asthma.

In asthmatic mice or bronchial epithelial cells (BEAS‐2B), TGF‐β1 easily induces EMT, which helps airway remodelling and is associated with the severity and progression of the disease.^26^ There is increasing evidence that DEK promotes the development of EMT by increasing the levels of mesenchymal marker proteins (such as vimentin) and decreasing the levels of epithelial marker proteins (such as E‐cadherin).^5^The down‐regulation of E‐cadherin in epithelial cells will lead to reduced cell‐to‐cell contact, resulting in increased cell permeability to allergens and increased sensitivity to injury.^27^ In addition, α‐SMA expressed in the epithelial cells exerts pressure on fibres and intermediate filament vimentin to produce extracellular matrix through secretion of fivronectin and collagen, thus promoting EMT and airway remodelling.^27^ Here, we surprisingly found that during TGF‐β1‐mediated EMT, overexpression of DEK in BEAS‐2B cells down‐regulated the expression of E‐cadherin and up‐regulated vimentin and α‐SMA. However, DEK silencing inhibited the phenotype of EMT. These results are consistent with the previous study that DEK proteins promoted EMT, metastasis and DNA damage in cancers.^28^ In addition, cells overexpressing DEK induced the expression of TGF‐β1 in the BEAS‐2B cells, as well as increased Smad2/3 and Smad 4 phosphorylation. Therefore, we reported for the first time that DEK induced the production of TGF‐β1 in human bronchial epithelial cells and the downstream canonical Smad signalling pathways may be involved in this process.

In addition to the classical Smad signalling pathway, DEK may also regulate other non‐classical signalling pathways involved in the EMT process mediated by TGF‐β1 in BEAS‐2B cells, including ERK1/2, JNK, p38 MAPK, PI3K/AKT/mTOR (non‐Smad signalling pathway). PI3K/AKT/mTOR inhibition has been shown to be closely related to airway remodelling.^29^ In this study, we showed that silencing DEK significantly attenuated the activation of the PI3K/AKT/mTOR pathway, indicating that DTA‐64 could reduce the development of allergic asthma through the PI3K/AKT/mTOR pathway.

In asthmatic mice, the ERK activity in the lung is significantly higher than that in normal mice,^30^, ^31^ and p38 MAPK and JNK inhibitors (SB‐203580 and SP‐600125) regulate TGF‐β1‐induced EMT in pulmonary epithelial cells.^32^, ^33^ Given the important role of the MAPK pathway in airway remodelling, we hypothesize that silencing DEK may inhibit EMT inhibition via MAPK pathway during airway remodelling of severe asthma. As expected, our data indicate that overexpression or knockdown of DEK significantly increased or inhibited the activation of p38 MAPK, ERK1/2 and JNK after TGF‐β1 stimulation, respectively. These results are consistent with previous report that silencing DEK inhibits p‐ERK1/2 expression, thereby inhibiting the proliferation and angiogenesis of cholangiocarcinoma cells.^34^ Therefore, the inhibitory effect of DEK silencing observed in TGF‐β1‐mediated EMT may be attributed to the MAPK signalling pathway in the airway epithelium.

The limitation of the manuscript is the use of only one human bronchial epithelial cell line. It would be necessary to verify the results in other cell lines (such as 16HBE or smooth muscle cells) in the future.

**FIGURE 7:**
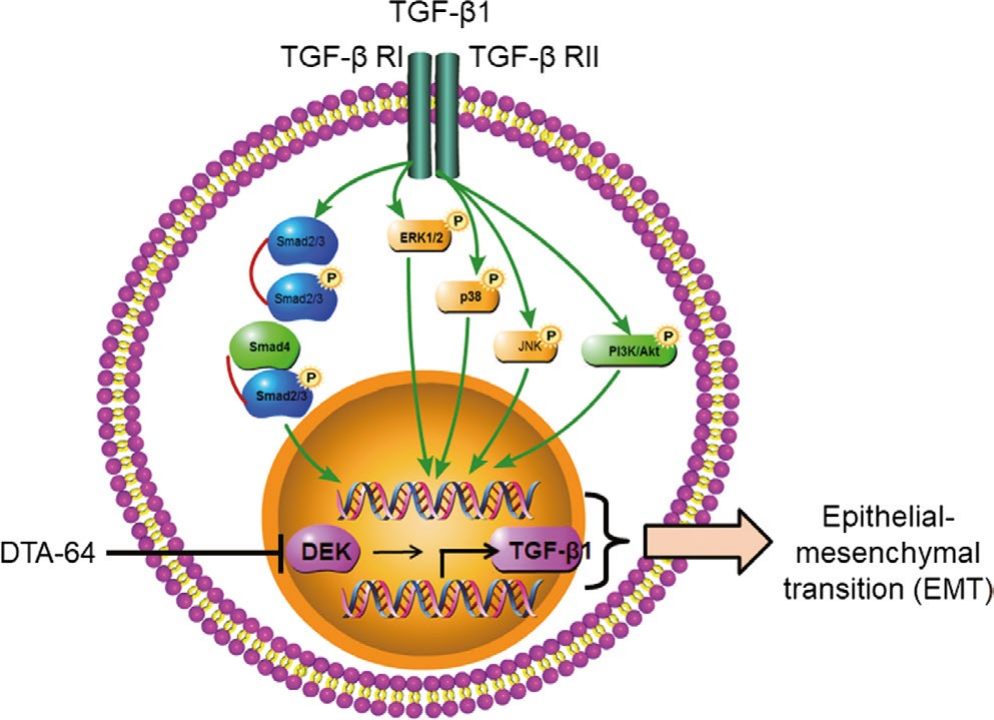
Schematic diagram for the signalling pathways related to the EMT and airway remodelling in response to TGF‐β1. TGF‐β1 binds to its ligands, a heterotetrameric receptor complex of two type I (TGF‐βRI) and type II receptors (TGF‐βRII), which then actives Smad family members, followed by trans‐locating into the nucleus to modulate target genes. This is often termed as canonical Smad signalling pathway. TGF‐β1 also activates Smad‐independent signalling pathways, such as p38 MAPK, JNK, ERK1/2 and PI3K/ AKT/mTOR. DEK may induce expression of TGF‐β1, which are secreted into the extracellular to amplify TGF‐β1 activity by autocrine and paracrine mechanisms

In summary, overexpression of DEK in the BEAS‐2B cells could co‐stimulate EMT process, through Smad‐dependent signalling pathways and Smad‐independent signalling pathways, such as p38 MAPK, JNK, ERK1/2 and PI3K/AKT/mTOR. This may be through the mechanism that DEK induces expression of TGF‐β1, which is then secreted into the extracellular to amplify TGF‐β1 signalling by autocrine and paracrine mechanisms (Figure 7).

To our best knowledge, we evaluated for the first time the effect of DEK‐targeted aptamer (DTA‐64) on inflammation and EMT in asthmatic mice and human bronchial epithelial cells. The results showed that inhibition of DEK significantly alleviated the bronchial EMT process both in vivo and in vitro, and balanced the Th1/Th2/Th17 immune response in asthma model mice. These effects may be mediated through NF‐κB, MAPK and PI3K signalling pathways. These findings partially suggest that targeting DEK may be a potential therapeutic strategy for allergic airway remodelling, and however, the clinical application needs to be further studied.

## CONFLICT OF INTEREST

All authors declare no competing interests.

## AUTHOR CONTRIBUTION

**Yilan Song:** Data curation (equal); Formal analysis (equal); Methodology (equal); Software (equal); Writing‐original draft (equal). **Zhiguang Wang:** Data curation (equal); Formal analysis (equal); Methodology (equal); Software (equal); Writing‐original draft (equal). **Jingzhi Jiang:** Data curation (equal); Formal analysis (equal); Methodology (equal); Software (equal). **Yihua Piao:** Data curation (equal); Formal analysis (equal); Methodology (equal). **Li Li:** Data curation (equal); Formal analysis (equal); Methodology (equal). **Chang Xu:** Data curation (equal); Formal analysis (equal); Methodology (equal). **Hongmei Piao:** Data curation (equal); Formal analysis (equal); Methodology (equal). **Liangchang Li:** Conceptualization (equal); Validation (equal); Writing‐review & editing (equal). **Guanghai Yan:** Conceptualization (equal); Funding acquisition (equal); Writing‐review & editing (equal).

## Data Availability

The data that support the findings of this study are available from the corresponding author upon reasonable request.
