# Corrigendum

**DOI:** 10.1111/jcmm.17487

**Published:** 2022-08-02

In Yilan Song et al.,^1^ the image for Vimentin of TGF‐beta1‐NC group in Figure 5F contains error during image preparation. The correct figure is shown below. The authors confirm all results and conclusions of this article remain unchanged. The authors confirm that there are no conflicts of interest.

**FIGURE 5:**
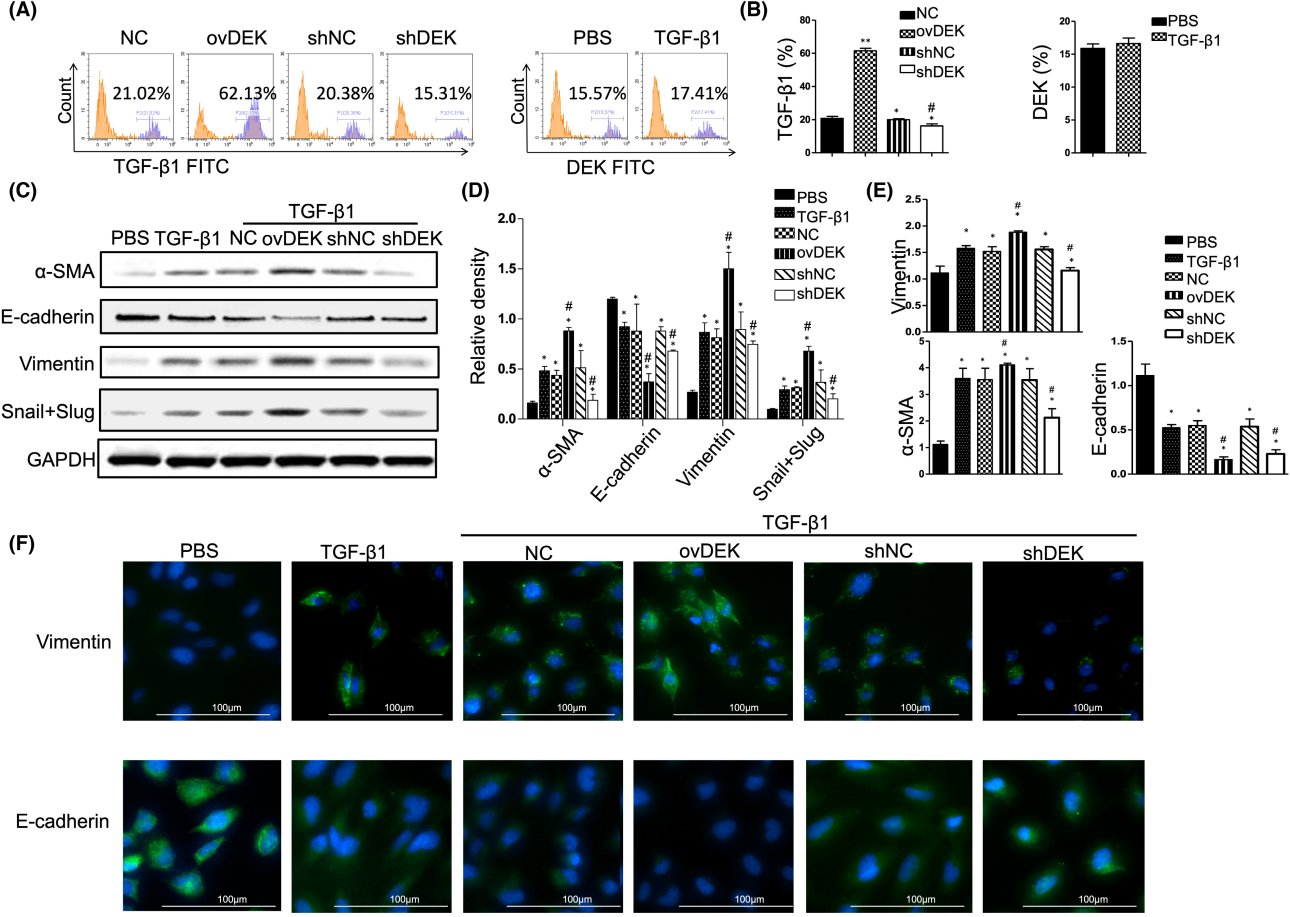
DEK positively regulates the progression of EMT in human bronchial epithelial cell. (A) Flow cytometry analysis of TGF‐β1 expression in the BEAS‐2B cells with ovDEK or shDEK (right panel) and DEK expression after treating with TGF‐β1 (left panel). (B) Relative level of TGF‐β1 and DEK. (C) The DEK, α‐SMA, vimentin, E‐cadherin and Snail + Slug were detected after TGF‐β1 treatment using Western blotting. (D) Relative density of above proteins. (E) mRNA levels of α‐SMA, vimentin and E‐cadherin were detected. (F) Immunofluorescence was performed for analysis of E‐cadherin and vimentin in the BEAS‐2B cells. NC, negative control cells; ovDEK, cells transfected with DEK; shNC, cells transfected with scrambled shRNA; shDEK, cells transfected with DEK shRNA.*p < 0.05, vs control group. ^#^ *p* < 0.05, ovDEK vs TGF‐β1 + NC group; shDEK v sTGF‐β1 + shNC group.
